# Prostate cancer clinical trials in low- and middle-income countries

**DOI:** 10.3332/ecancer.2023.1629

**Published:** 2023-11-13

**Authors:** Sattam A Halaseh, Amro Al-Karadsheh, Deborah Mukherji, Abdelrahman Alhjahaja, Ala’a Farkouh, Akram Al-Ibraheem, Ibrahim Abu Gheida, Sultan Al-Khateeb, Humaid Al-Shamsi, Mohammed Shahait

**Affiliations:** 1 Urology Department, Torbay Hospital, Torbay and South Devon NHS Foundation Trust, Newton Rd, Torquay TQ2 7AA, UK; 2 General Medicine, Lincoln County Hospital, United Lincolnshire Hospitals NHS Trust, Greetwell Rd, Lincoln LN2 5QY, UK; 3 Hematology/Oncology Division, Department of Internal Medicine, American University of Beirut Medical Center, Riad El Solh, Beirut 1107 2020, Lebanon; 4 The Hashemite University, Damascus Hwy, Zarqa 453J+5C5, Jordan; 5 American Center for Reproductive Medicine, Cleveland Clinic, 10681 Carnegie Ave, Cleveland, OH 44106, USA; 6 Department of Nuclear Medicine and PET/CT, King Hussein Cancer Centre, Queen Rania Al Abdullah Street, Amman 11941, Jordan; 7 Cleveland Clinic Abu Dhabi LLC, Al Marayyah Island, PO Box 112412, Abu Dhabi, UAE; 8 King Faisal Specialist Hospital & Research Center, PO Box 3354, Riyadh 11211, Kingdom of Saudi Arabia; 9 Burjeel Cancer Institute/VPS Oncology UAE, 28th Street, Mohammed Bin Zayed City Abu Dhabi, PO Box 92510, UAE; 10 Surgery Department, Clemenceau Medical Center, Dubai Healthcare City Phase 2 - Al Jaddaf, Dubai, UAE

**Keywords:** prostate cancer, developing countries, healthcare disparities, cost of illness, low middle-income countries

## Abstract

**Background:**

Prostate cancer is the second most common form of cancer and a leading cause of cancer-related death in men. In an era of evidence-based medicine, clinical trials play a critical role, and adherence to best practices is crucial in managing complicated and non-communicable diseases, such as prostate cancer. For this reason, extrapolating research conducted in high-income countries (HICs) to low-middle-income countries (LMICs) may lead to incorrect findings or treatment plans for patients in these areas. Unfortunately, clinical trials in LMICs face several challenges in terms of design, funding and recruitment. This study aimed to examine clinical trials on prostate cancer in LMICs, including the scope of these trials, the type of interventions being tested and funding sources.

**Methods:**

A search of the Cochrane Library Controlled Trials Registry was conducted between January 2010 and June 2021 using keywords including: ‘prostate cancer’, ‘prostate adenocarcinoma’ and ‘prostate tumour’). The trials were classified into either HICs or LMICs based on the World Bank Atlas classification. A descriptive analysis was performed to determine the characteristics of the trials.

**Results:**

A total of 3,455 clinical trials for prostate cancer have been conducted globally, with 542 (15.68%) conducted LMICs. Most of these trials (89%) were registered in upper-middle-income countries, with none being conducted in low-income countries. The majority of trials were prospective studies (98.1%), with 65.2% being randomised and 57% being phase III. Of the trials, 48.4% aimed to recruit fewer than 500 participants. The main source of funding was pharmaceutical companies in 78.1% of the cases, followed by institutional funds (16.1%) and public funds (5.8%). At the time of the search query, 74.6% of the trials were inactive, with 37% completed, 5% terminated due to insufficient funding and 75% terminated due to medical inefficacy or poor accrual. The majority of trials (88.2%) were interventional, with only 6% focusing on screening and prevention, and 2% designed for palliative care.

**Conclusion:**

This study sheds light on the challenges faced in conducting clinical trials for prostate cancer in LMICs. The findings underline the need for improved support from international organisations and pharmaceutical companies to bridge the gaps in prostate cancer research and facilitate collaboration between researchers in LMICs and other countries.

## Background

Genitourinary malignancies such as prostate, renal, bladder, urethral, testicular and penile cancers represent almost 12.7% of newly diagnosed cancers in the world and almost 7.9% of cancer-related deaths globally. In 112 nations, prostate carcinoma was the most prevalent cancer diagnosed in men, followed by lung, colorectal and liver cancers [1]. In 2020, prostate cancer accounted for 375,000 deaths globally, with 1,400,000 new cases being diagnosed [2].

The regional incidence rates of prostate cancer range from 6.3% to 83.4% per 100,000 men, with the highest rates in Western Europe and Northern America and the lowest in Asia and Northern Europe. Regional patterns of death rates did not correspond to those of incidence, with the highest fatality rates in the Caribbean, sub-Saharan Africa and Micronesia/Polynesia. In 48 countries, including sub-Saharan Africa, the Caribbean, Central and South America (such as Ecuador, Chile and Venezuela) and Sweden, prostate cancer is the leading cause of cancer-related mortality among males [1].

In the era of evidence-based medicine, in which there is a growing emphasis on adherence to best practice guidelines, it is crucial to note that research conducted on a specific group of subjects may not be representative or applicable to other populations [3]. As a result, extrapolating from research undertaken by high-income countries (HICs) on complicated noncommunicable diseases, such as cancer or trauma, is challenging and may lead to impractical application in the context of limited resources [4], leading to inappropriate diagnostic approaches or treatment plans. This is crucial in establishing recommendations for best practices in low-middle-income country (LMIC) [5].

However, multilayer complexities hinder any efforts to design, fund and recruit clinical trials in LMIC countries [6]. As the management of locally advanced prostate cancer is evolving rapidly, there is limited data on the efficacy and feasibility of different novel therapies in LMICs, for example [5].

In this study, we sought to investigate the clinical trials of prostate cancer conducted in LMICs to better understand the scale of these trials, the type of intervention and funding sources. The results of this study may help international societies and pharmaceutical companies existing gaps in prostate cancer research and explore avenues for collaboration with researchers from LMIC.

## Methods

**Figure 1.**
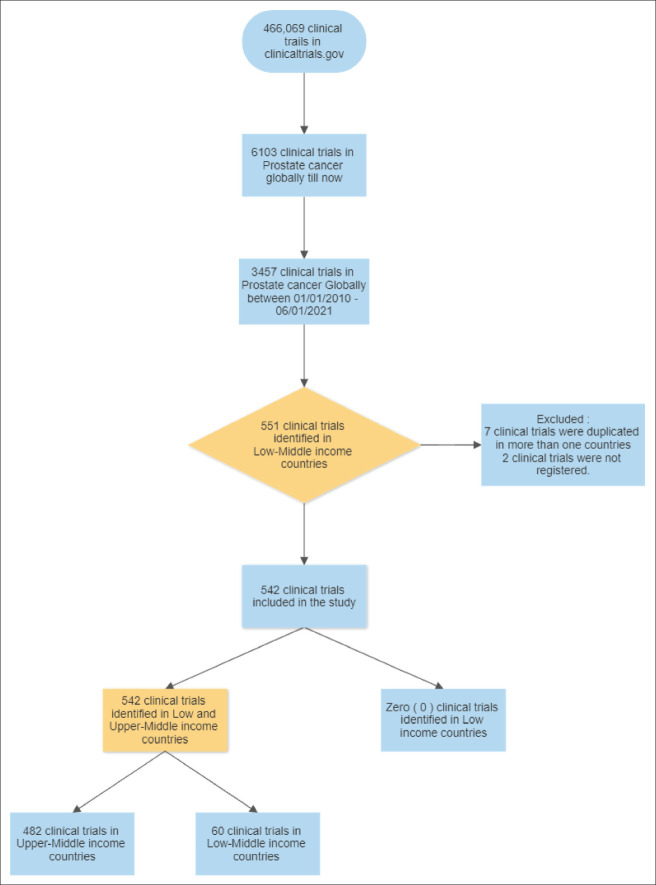
Evidence acquisition flow chart.

A comprehensive search of the Cochrane Library Controlled Trials Registry was performed to include clinical trials conducted between January 2010 and June 2021. The search keywords included prostate cancer ‘prostate cancer’, ‘prostate adenocarcinoma’, and ‘prostate tumour’, and prostate tumor (Figure 1). All clinical trials on prostate cancer screening, diagnosis, management and follow-up were included. The authors conducted the search. The search query was performed on 20 March 2022.

The following data were extracted from each study: study year, country, date of study initiation, date of study completion, study type, primary purpose, time perspective, allocation, observational model, phase, intervention model, masking, type of intervention, control arm, sample size, active or inactive trial, reason for ending the trial, funding type and number of institutions (single or multicentre).

The World Bank Atlas classifies countries into low-income, lower-middle-income, upper-middle-income and high-income economies [7] (Table 1). For the analysis, the LMIC was used to represent countries with low- and middle-income economies. Descriptive statistics were used to analyse the results (Table 2).

## Results

A total of 3,455 prostate cancer trials have been conducted globally. 542 (15.68%) prostate clinical trials were identified in LMIC. Seven trials were duplicates and excluded from the upper-middle-income countries. Most of these trials were registered in upper-middle-income countries 489 (89%), and none were identified in low-income countries (Figure 1).

The majority of prostate cancer clinical trials in LMIC (67.47%) were conducted after 2010. Most of these trials were prospective (98.1%), randomised (65.2%), phase III (57%) and planned to recruit <500 subjects (48.4%). The primary funding source for these trials was pharmaceutical companies (78.1%), followed by institutional funds (16.1%) and public funds (5.8%). Most of the trials were multicentre trials (83.39%). Refer to Table 2 for further details.

At the time of search query, 74.6% were labelled inactive trials. 37% were completed trials, with 5% terminated to conclusion of the trial, 25% due to insufficient funding and 75% terminated due to medical inefficacy or poor accrual.

Prostate cancer clinical trials in LMIC were mainly interventional (88.2%), using medication. Only 6% of trials focused on screening and prevention, while (2%) of trials were designed for palliative intent.

## Discussion

**Figure 2.**
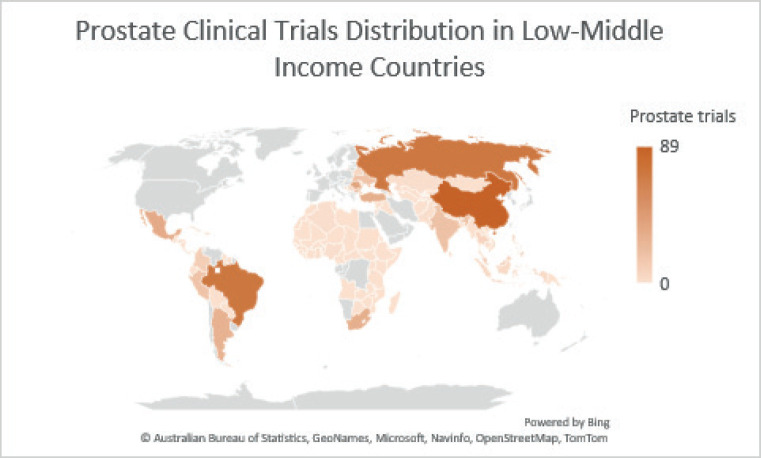
Prostate clinical trials distribution in LMICs.

This study aimed to provide an in-depth understanding of the current status of prostate cancer clinical trials in LMICs. Of the total 3,455 global clinical trials for prostate cancer, only 542 were conducted in middle-income countries and none were conducted in low-income countries (Figure 2). Most of these trials were funded by pharmaceutical companies and involved prospective multicenter recruitment of fewer than 500 patients. Furthermore, most trials were inactive (74.6%).

Our study yielded findings consistent with prior research in the field, underscoring the insufficiency of clinical trials focused on prostate cancer in LMICs relative to the disease burden in these regions. This aligns with the results of a retrospective study that revealed a higher number of phase III trials related to oncology in HICs than the disease burden, as prostate cancer is the fourth most common cancer in several regions of LMICs, including Africa. Moreover, none of the prostate cancer clinical trials have been conducted in low-income countries, despite evidence suggesting that the incidence of urological diseases may be higher than that currently reported [3, 6, 8]. These findings highlight the urgent need for further clinical trials to address the growing burden of prostate cancer in LMICs.

Moreover, the evidence generated from trials conducted in HICs may not apply to LICs because of lack of resources, different treatment sequences, accessibility of care and different genomic make-up of the tumours [4, 9]. Cancer treatment toxicities and adverse effects, including adrenal hormone suppression and radiotherapy, are more pronounced in certain races. Additionally, there are differences in treatment responses across races [10].

Interestingly, several studies have also indicated that the availability of resources and funding is a crucial driver of clinical trial activity rather than the population's needs [3]. The high cost of oncological clinical trials is often more significant than the annual gross national income per capita, making it a significant challenge to conduct trials in these regions [8]. Our study also highlights the role of pharmaceutical companies in funding prostate cancer clinical trials, with almost three-quarters of the funding from these companies.

International and local institutions must intervene to counteract this large gap and prevent possible future bias in the results of clinical trials. Other research has emphasised the need for proper management of funding in LMICs, for example, the need to ensure that the work of the National Institution of Health is distributed more equitably to support clinical trials focused on a range of diseases beyond infectious diseases and regions other than South Africa [11].

Additionally, studies have found that clinicians in LMICs face a significantly higher workload than do those in other countries, making it almost impossible to find time to conduct clinical trials. Furthermore, trainees receive limited teaching regarding critical appraisal and research methodology [12, 13], further exacerbating the challenges of conducting clinical trials in LMICs. These findings underscore the need for increased investment in training programs for healthcare providers in LMICs to support their participation in clinical trials and other research initiatives [14].

An additional challenge in conducting clinical trials in LMIC is participant recruitment. A nationwide survey from Jordan, a middle-income country, included 3,196 participants and reported that less than 25% were willing to participate in clinical trials. Only 21.8% of the participants knew what the clinical trials were, with socioeconomic factors being a significant determinant of knowledge and willingness to participate [15]. This study highlights the importance of raising awareness and providing proper patient education regarding clinical trials in LMICs.

In addition to the challenges related to funding, training and recruitment, studies have also revealed disparities in the publication of clinical trials conducted in LMICs despite their positive value. Clinical trials in LMICs that demonstrate significantly prolonged survival with significantly lower funding are often published in low-impact journals. Clinical trials in HICs that cost hundreds of thousands of dollars and show only marginal prolongation of survival have been published in high-impact journals. This disparity in publication may contribute to the under-representation of LMICs in prostate cancer, creating what is often referred to as ‘publication prejudice’ [14].

Several journals have attempted to decrease the gap in this area by providing more opportunities for LMICs to present their results and data. For example, the American Society of Oncology's Journal of Global Oncology and Cancer has put more effort into decreasing the publication gap by providing more opportunities for LMICs to publish their research [16]. This is an essential step toward ensuring that clinical trials conducted in LMICs receive the recognition they deserve and contribute to the advancement of medical knowledge worldwide.

## Conclusion

This study sheds light on the challenges faced in conducting clinical trials for prostate cancer in LMICs. The findings underline the need for improved support from international organisations and pharmaceutical companies in order to bridge the gaps in prostate cancer research and facilitate collaboration between researchers in LMICs and other countries.

## Conflicts of interest

The authors declared that they have no competing interests.

## Funding

The authors did not receive any external sources of funding.

## Author contributions

Primary author: Sattam A. Halaseh, Mohammed Shahait and Amr Al-Karadsheh. All authors gave substantial contributions to the conception or design of the work; or the acquisition, analysis or interpretation of data for the work; drafting the work or revising it critically for important intellectual content; and final approval of the version to be published. They agree to be accountable for all aspects of the work in ensuring that questions related to the accuracy or integrity of any part of the work are appropriately investigated and resolved.

## Figures and Tables

**Search strategy**

**Topic:** Prostate cancer clinical trials in low-middle income countries

**Date range:** 01/01/2010–06/01/2021

**Language:** English

**Human only, globally**

**Search strategy terms**

Prostate cancer

TS = ((Prostat* AND (cancer* OR carcinoma* OR adenocarc*)))

https://clinicaltrials.gov/search?cond=PROSTATE%20CANCER&start=2010-01-01_2021-06-01

**Total number of clinical trials globally: 3,457**

**Total number of clinical trials in LMICs = 551**

**(Seven duplicated studies, two not registered).**

**Colour gradient**

**Grey colour:** Indicates areas with the least activity, intensity or lowest data values.

**Light orange:** Represents areas with below-average values.

**Orange:** Signifies areas with median or average values.

**Deep orange:** Marks areas with the highest activity, intensity or peak data values.

**Table 1.** Prostate clinical trials distribution heat map in LMICs.

Country	Prostate trials	Country	Prostate trials	Country	Prostate trials	Country	Prostate trials	Country	Prostate trials	Country	Prostate trials	Country	Prostate trials
Afghanistan	0	South Sudan	0	El Salvador	0	Pakistan	1	Azerbaijan	0	Kazakhstan	1	Thailand	7
Burkina Faso	0	Sudan	0	Eswatini	0	Papua New Guinea	0	Belarus	6	Kosovo	0	Toga	0
Burundi	0	Syrian Arab Republic	0	Ghana	1	Philippines	6	Bosnia and Herzegovina	1	Lebanon	3	Turkey	31
Central African Republic	0	Togo	0	Haiti	0	Samoa	0	Botswana	0	Libya	0	Turkmenistan	0
Chad	0	Uganda	0	Honduras	0	São Tomé and Principe	0	Brazil	72	Malaysia	9	Tuvalu	0
Congo, Dem. Rep	0	Yemen, Rep.	0	India	18	Senegal	0	Bulgaria	18	Maldives	0		
Eritrea	0	Angola	0	Indonesia	3	Solomon Islands	0	China	89	Marshall Islands	0		
Ethiopia	0	Algeria	1	Iran, Islamic Rep	2	Sri Lanka	0	Colombia	11	Mauritius	0		
Gambia, The	0	Bangladesh	0	Kenya	0	Tanzania	0	Costa Rica	1	Mexico	33		
Guinea	0	Belize	0	Kiribati	0	Tajikistan	0	Cuba	1	Moldova	5		
Guinea-Bissau	0	Benin	0	Kyrgyz Republic	0	Timor-Leste	0	Dominica	0	Montenegro	2		
Korea, Dem. People's Rep	0	Bhutan	0	Lao PDR	0	Tunisia	2	Dominican Republic	0	Panama	3		
Liberia	0	Bolivia	0	Lesotho	0	Ukraine	17	Equatorial Guinea	0	Paraguay	0		
Madagascar	0	Cape Verde	0	Mauritania	0	Uzbekistan	0	Ecuador	0	Peru	17		
Malawi	0	Cambodia	0	Micronesia, Fed. Sts.	0	Vanuatu	0	Fiji	0	Romania	38		
Mali	0	Cameroon	0	Mongolia	0	Vietnam	2	Grenada	0	Russian Federation	71		
Mozambique	0	Comoros	0	Morocco	0	West Bank and Gaza	0	Guatemala	0	Serbia	9		
Niger	0	Congo, Rep.	0	Myanmar	0	Zambia	0	Guyana	0	South Africa	31		
Rwanda	0	Côte d'Ivoire	0	Nepal	0	Zimbabwe	0	Iraq	0	St. Lucia	0		
Sierra Leone	0	Djibouti	0	Nicaragua	0	Argentina	29	Jamaica	1	St. Vincent and the Grenadines	0		
Somalia	0	Egypt, Arab Rep.	6	Nigeria	1	Armenia	0	Jordan	0	Suriname	0		

**Table 2.** Descriptive analysis of LMIC trials.

	Low income	Low-middle income	Upper middle income
Study date			
1990–2000	0	0	8
2001–2010	0	3	163
2011–2020	0	37	308
>2020	0	2	14
Study type			
Observational	0	8	55
Interventional	0	34	438
Primary purpose			
Treatment	0	28	406
Medical	0	2	3
Surgical	0	0	0
Diagnostic	0	3	20
Prevention	0	4	5
Palliative	0	2	9
Outcome	0	2	51
Study design			
Prospective	0	40	485
Cross sectional	0	2	0
Retrospective	0	0	8
Status			
Active	0	10	126
Inactive	0	32	367
Sample size			
<500	0	27	232
500–1,000	0	7	91
>1,000	0	8	170
Funding			
Public	0	14	17
Pharma	0	24	394
Institute	0	4	82
Centre			
Single centre	0	17	66
Multi centre	0	24	428
